# ESUR female pelvis group approach to cystic female pelvic lesions

**DOI:** 10.1186/s13244-025-02174-4

**Published:** 2026-02-16

**Authors:** Olivera Nikolić, Lucia Manganaro, Milagros Otero Garcia, Stephanie Nougaret, Isabelle Thomassin-Naggara, Refky Nicola, Nemanja Maletin, Charis Bourgioti

**Affiliations:** 1 Faculty of Medicine, University of Novi Sad, Novi Sad, Serbia; 2 Center of Radiology, University Clinical Center of Vojvodina, Novi Sad, Serbia; 3 Department of Radiological, Oncological and Pathological Sciences, Sapienza University of Rome, Rome, Italy; 4 Hospital Universitario de Vigo Alvaro Cunqueiro, Vigo, Spain; 5 Department of Radiology Montpellier Cancer Institute, Montpellier France PINKCC lab, U1194, Montpellier Cancer Research Institute, Montpellier, France; 6 APHP, Radiology Imaging and Interventional Radiology Specialized Department (IRIS), Tenon Hospital, Public Hospital of Paris, Paris, France; 7 INSERM UMR S 938, CRSA Saint-Antoine Hospital, Sorbonne University, Paris, France; 8 MS DO, SUNY Upstate Medical University, Syracuse, NY USA; 9 First Department of Radiology, School of Medicine, National and Kapodistrian University of Athens, Aretaieion Hospital, Athens, Greece

**Keywords:** Pelvic, Ovarian, Non-ovarian, Cystic, MRI

## Abstract

**Abstract:**

Cystic female pelvic lesions, whether of ovarian or non-ovarian origin, are prevalent in routine clinical practice, with the majority originating from gynaecological (ovarian) structures, ranging from functional cysts to malignant ovarian tumours. Despite the fact that we encounter these lesions in the course of our routine clinical work, arriving at an accurate diagnosis can often prove challenging due to the overlap of imaging appearances. Ultrasound is the primary imaging modality for the evaluation of most cystic female pelvic lesions, while MRI serves as a problem-solving tool. In cases that are more complex or equivocal, pelvic MRI proved to be particularly useful due to its superior soft tissue resolution, multiplanar imaging capability and non-invasive nature. In order to make an accurate diagnosis, it is crucial to have a comprehensive understanding of pelvic topographic anatomy, be familiar with possible differential diagnoses and include all relevant clinical data. The classification of ovarian cystic lesions was undertaken using the O-RADS MRI risk stratification system, which provides standardised language for communication between radiologists and clinicians. The objective of this review is to illustrate the spectrum of typical MRI characteristics of different cystic female lesions of both ovarian and non-ovarian origin, with the emphasis on differential diagnoses. The review includes tables with MRI appearances on T2, T1, DWI sequences and postcontrast tomograms. To facilitate the learning process, schematic representations of MRI appearances of ovarian lesions have been incorporated.

**Critical relevance statement:**

MRI diagnosis of various ovarian and non-ovarian cystic female pelvic lesions and their differential diagnosis.

**Key Points:**

- The diagnosis of cystic female pelvic lesions can be challenging due to the overlapping imaging characteristics exhibited by these lesions.
- Discrimination between ovarian and non-ovarian lesions is of paramount importance, given the existence of marked discrepancies in both prognosis and management.
- If the lesion is of ovarian origin, the O-RADS MRI risk stratification system should be implemented in order to ascertain the risk of malignancy.

**Graphical Abstract:**

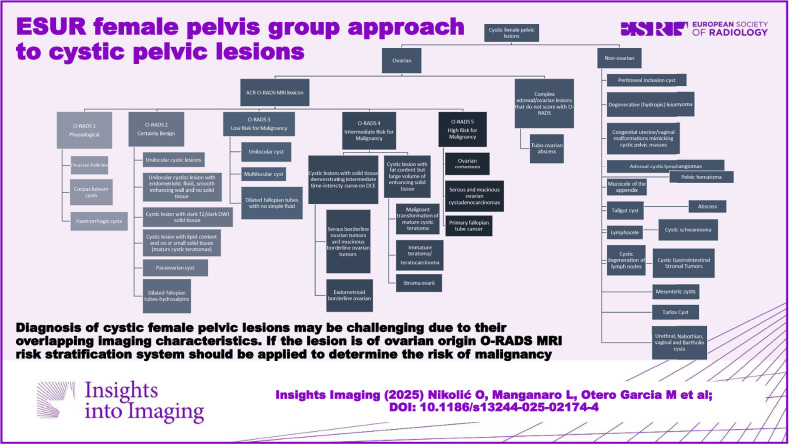

## Introduction

Cystic female pelvic lesions, whether of ovarian or non-ovarian origin, are prevalent in routine clinical practice, with the majority arising from gynaecological (ovarian) structures. Different types of cystic pelvic lesions may exhibit similar radiological characteristics, which can sometimes complicate imaging evaluation and reduce diagnostic accuracy. Ovarian cystic pelvic lesions encompass functional follicles, benign ovarian cysts and malignant ovarian lesions.

Furthermore, a variety of mimics of ovarian cystic lesions can be found in the pelvis. Cystic pelvic lesions that are separated from the ovary are referred to as non-ovarian [1].

Ultrasound is the primary imaging modality for the evaluation of most cystic female pelvic lesions, while MRI serves as a problem-solving tool. Pelvic MRI has been shown to be particularly useful in more complex or equivocal cases due to its superior soft tissue resolution and excellent tissue characterisation, multiplanar imaging capability and non-invasive nature [2].

An accurate diagnosis of cystic female pelvic lesions requires a thorough understanding of pelvic topographic anatomy and a comprehensive knowledge of the wide spectrum of possible differential diagnoses. In order to enhance diagnostic confidence, it is essential to consider a comprehensive patient medical history, relevant laboratory findings, and imaging identification of the lesion's anatomical location, which, on occasion, presents a challenge. Magnetic resonance imaging (MRI) plays a crucial role in the evaluation of pelvic masses, particularly in determining whether a lesion is of adnexal origin or arises from another pelvic structure. A number of imaging signs have been described with the aim of assisting in identifying the anatomical origin of such masses [3–5].

The objective of this article is to describe and analyse the imaging characteristics in MRI that allow differential diagnosis between cystic masses of adnexal and non-adnexal origin, and to provide guidance for the differential diagnosis of ovarian cystic lesions following the ovarian-adnexal reporting and data system (O-RADS) MRI risk stratification system.

## Cystic female pelvic lesions

**Fig. 1:**
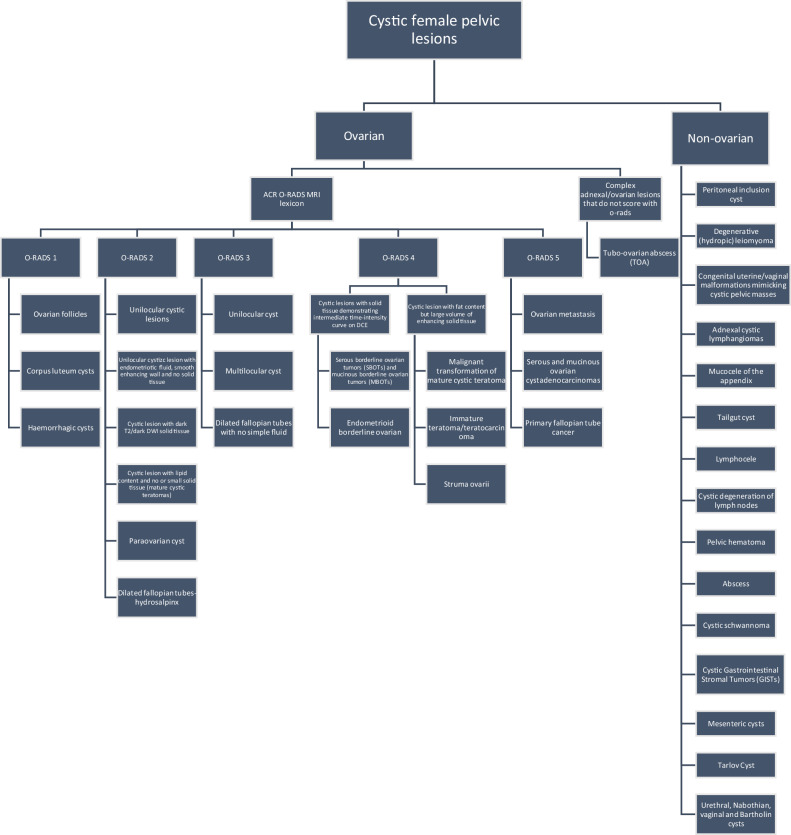
Classification of cystic female pelvic lesions

Cystic female pelvic lesions can be categorised into two broad classifications: those of ovarian and non-ovarian origin. Each of these categories can then be further subdivided into benign and malignant lesions (Fig. 1).

Lesions can be classified as purely cystic, mixed (cystic with solid tissue), or purely solid. Cystic lesions are defined as fluid-filled structures that may be unilocular or multilocular [4]. A comprehensive delineation of the terms and definitions for signal intensity (SI), physiologic finding vs lesion, and fluid vs solid component lies beyond the scope of this paper and can be found in the ACR O-RADS MRI lexicon [6].

It is evident that radiologists encounter difficulties in reporting cystic pelvic lesions largely attributable to the overlap in their imaging characteristics. The O-RADS MR system is a way to assess the risk of malignancy of any ovarian lesion based only on MR features [5]. O-RADS MRI risk score does not apply to lesions found to be non-ovarian in origin. It is important to acknowledge that a lesion of specific pathology may be categorised in a different O-RADS category based on its unique imaging characteristics.

O-RADS classification system can be applied to the majority of cystic adnexal lesions encountered in clinical practice, as outlined below:

### O-RADS MRI 1: cystic physiological ovarian lesions

The O-RADS MRI score 1 is assigned when the ovaries are normal; in the premenopausal population, functional ovarian ‘lesions’ including follicles, haemorrhagic cysts and corpus luteum measuring 3 cm or less are considered a normal finding.

#### Ovarian follicles

**Fig. 2:**
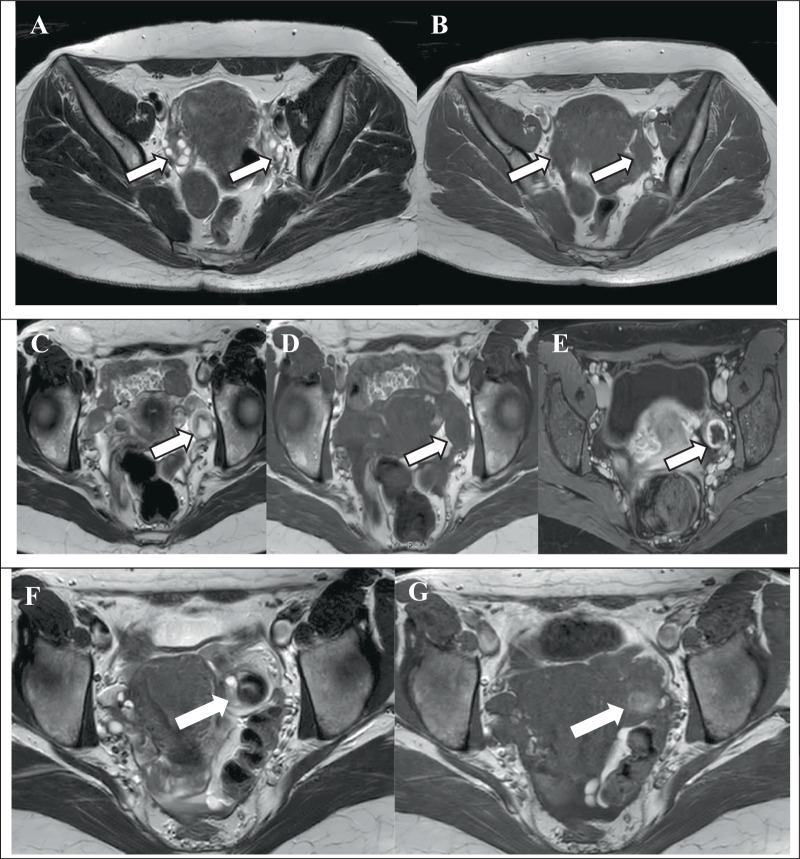
O-RADS MRI 1: cystic physiological ovarian lesions. Ovarian follicles: axial T2-weighted (**A**) image shows hyperintense follicles (white arrows), and axial T1-weighted image (**B**) shows hypointense follicles of both ovaries (white arrows). Corpus luteum cyst: axial T2-weighted image (T2WI) (**C**) and axial T1-weighted image (**D**) showing the cyst with a thickened wall (white arrow). Post-contrast T1-weighted fat-suppressed (T1Wfs) (**E**) image demonstrating peripheral rim enhancement of the cyst (white arrow). Haemorrhagic ovarian cysts: axial T2WI (**F**) shows a haemorrhagic ovarian cyst with dense internal fluid (white arrow), while axial T1-weighted image (**G**) demonstrates a hyperintense signal of the cyst due to haemorrhagic content (white arrow)

As is the case with all simple cysts, ovarian follicles appear hyperintense on T2WI, hypointense on T1-weighted images (T1WI), measure less than 3 cm and exhibit peripheral post-contrast enhancement (Table 1 and Fig. 2).

**Table 1:** ORADS 1—MRI characteristics of ovarian follicles, corpus luteum and haemorrhagic cysts

Cystic lesion	T2W	T1W	+C T1W	DWI
Ovarian follicles			−	
Corpus luteum cyst			+ (rim)	
Haemorrhagic cysts				
Hyperacute				
Acute				
Early subacute				

#### Corpus luteum cysts

Corpus luteum cysts demonstrate analogous MRI characteristics to simple cysts, namely T1 and T2, yet demonstrate high DWI signal and intense rim enhancement of their thick wall following the administration of contrast (Table 1 and Fig. 2). Typically, these lesions are less than 3 cm in diameter and can be classified as either simple or complex, with the latter being characterised by the presence of haemorrhage. Other less common findings may include multifocality, bilaterality, and rupture, which can lead to hemoperitoneum-heterogeneous or T1 hyperintense ascites, along with a focal interruption of the cyst wall [7, 8]. A differential diagnosis of corpus luteum must consider other potential complex cystic lesions, including endometrioma or tubo-ovarian abscess (TOA). These must be considered within the appropriate clinical context, as outlined in the following text.

#### Haemorrhagic cysts

Haemorrhagic cysts may display varying MRI appearances depending on the stage of the haemorrhage (Table 1). These cysts typically resolve within eight weeks, which is a significant diagnostic consideration when differentiating them from endometriomas. Both haemorrhagic cysts and endometriomas demonstrate hyperintense signal on a T1-weighted fat-suppressed sequence, a finding attributed to the presence of blood (Fig. 2). The differential diagnosis is based on the higher and more homogeneous T1W SI of endometrioma (typically higher than fat on a non-fat-sat sequence) than haemorrhagic cyst. The presence of a fibrinous component within a haemorrhagic cyst can result in a mimicry of septations and/or mural nodules. Gadolinium injection demonstrates the absence of internal enhancement of these structures and is helpful to avoid any overdiagnosis.

### O-RADS MRI 2 (non-physiological lesions): adnexal lesions are almost certainly benign (PPV for malignancy of less than 0.5%)

The O-RADS 2 score is typically assigned to the following adnexal cystic lesions (Table 2):

**Table 2:** ORADS 2—MRI characteristics of cystic lesions

Cystic lesion	T2W	T1W	T1Wfs	DWI
Unilocular cystic lesions			-	
Endometrioma				
Ovarian cystadenofibroma			-	
Mature cystic teratomas (purely cystic)				
Mature cystic teratomas (complex)				
Paraovarian cysts			-	
Dilated fallopian tubes			-	

#### Unilocular cystic lesions

**Fig. 3:**
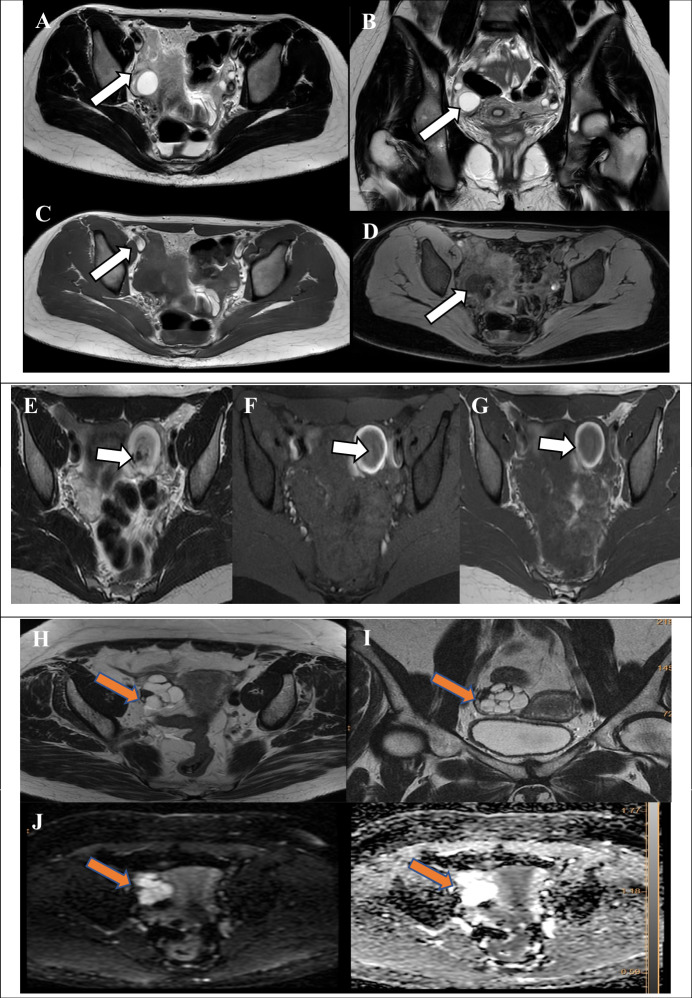

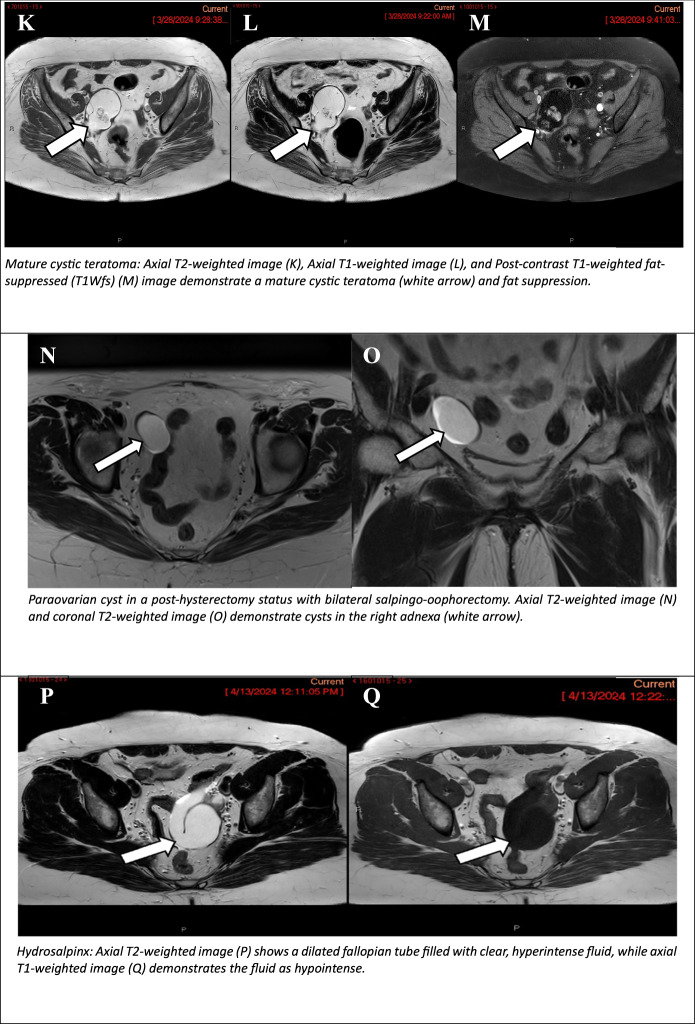
O-RADS MRI 2 (non-physiological lesions): adnexal lesions are almost certainly benign. Unilocular cystic lesion measuring approximately 4 cm. Axial T2WI (**A**) shows a hyperintense lesion, while coronal T2WI (**B**) confirms its location. Axial T1-weighted image (**C**) demonstrates hypointense follicles of the right ovary, and DIXON image (**D**) provides additional characterisation of the lesion (white arrows). Endometrioma: axial T2-weighted (**E**) image showing the “black dot sign” indicative of chronic haemorrhage (white arrow). Axial T1-weighted fat-suppressed (T1Wfs) (**F**) image demonstrating the “shading sign” (white arrow), which represents recurrent haemorrhage. Axial T1-weighted image (**G**) confirms high SI of the cystic content (white arrow). Ovarian cystadenofibroma: Axial T2WI shows a dark spot (**H**, **I** orange arrow) and thick septations within the mass. No diffusion restriction on high b-value DWI and ADC map (**J**). Mature cystic teratoma: Axial T2WI (**K**), Axial T1-weighted image (**L**), and Post-contrast T1-weighted fat-suppressed (T1Wfs) (**M**) image demonstrate a mature cystic teratoma (white arrow) and fat suppression. Paraovarian cyst in a post-hysterectomy status with bilateral salpingo-oophorectomy. Axial T2WI (**N**) and coronal T2WI (**O**) demonstrate cysts in the right adnexa (white arrow). Hydrosalpinx: axial T2WI (**P**) shows a dilated fallopian tube filled with clear, hyperintense fluid, while axial T1-weighted image (**Q**) demonstrates the fluid as hypointense

Unilocular cystic lesions devoid of wall enhancement or solid tissue, containing any type of fluid. A typical example is a large haemorrhagic cyst measuring ≥ 3 cm (Fig. 3) [9].

Unilocular cystic lesion with thin-walled enhancement and simple fluid, but no solid tissue. A typical example is a large follicular cyst measuring ≥ 3 cm.

#### Unilocular cystic lesion containing endometriotic fluid, a smooth enhancing wall and no solid tissue

Endometriotic fluid typically demonstrates a homogeneously hyperintense T1W signal with a corresponding T2W hypointense signal, a phenomenon referred to as the “T2 shading sign”. The shading sign is indicative of an MR finding of T2 shortening in an adnexal cyst that is hyperintense on T1W images. Furthermore, endometrioma is generally characterised by its unilocular nature and the presence of the so-called “T2 dark spot sign”. The T2 dark spot sign exhibits low sensitivity (36%), but high specificity (93%) for endometrioma, thus facilitating its differentiation from functional haemorrhagic cyst [10, 11]. The T2 dark spot sign is defined as a focal hypointense spot that can appear anywhere within the cyst, but not in the cyst wall. It represents a blood clot containing heavy concentrations of protein and iron due to chronic haemorrhage [12] (Fig. 3). This feature is instrumental in differentiating between an endometrioma and endometriotic hematosalpinx, as the latter does not exhibit this characteristic. Furthermore, multiloculated endometriomas or a hematosalpinx with endometriotic content are classified as O-RADS 2 lesions.

#### Cystic lesion with dark T2/dark DWI solid tissue

The most representative lesion in this category is cystadenofibroma, which typically manifests as a multilocular cystic mass with a T2-dark-SI solid component containing small cystic locules. In instances where the solid component of the lesion exhibits low T2 and low DWI SI, it is categorised as O-RADS 2 (Fig. 3) irrespective of the time-intensity curve (TIC) pattern that is evident on dynamic contrast-enhanced (DCE) MRI [13]. However, the presence of a prominent solid component with a higher T2 SI, diffusion restriction and strong enhancement with TIC type 3 raises the suspicion of malignancy.

#### Cystic lesion with lipid content and no or small solid tissue (mature cystic teratomas)

Fat (lipid) containing fluid appears hyperintense on T1W/T2W with loss of signal on fat-saturated sequence (T1FS) (Fig. 3). The presence of intravoxel fat within a cystic lesion detected on chemical shift T1W out-of-phase sequence (3DT1W DIXON sequence), even in the absence of noticeable macroscopic fat, still represents “lipid-containing fluid” (i.e. dermoid) [7]. The imaging findings are variable, and may consist of a purely cystic mass or a complex cystic mass containing a considerable amount of a solid component. A Rokitansky nodule is defined as a small, solid component (i.e. typically comprising a mixture of tissues, such as hair, teeth, bone or fat) that may demonstrate enhancement, yet it is not considered to be solid tissue (Fig. 3). Consequently, even in the presence of a Rokitansky nodule, the lesion is still classified as O-RADS 2. However, in cases where there is more solid tissue than is expected for a typical Rokitansky nodule demonstrating irregular borders or extracapsular growth, malignant transformation of the dermoid may be suspected, and the lesion is classified as O-RADS 4 [14, 15].

#### Paraovarian cyst

Paraovarian cysts are derived from the mesosalpinx. Benign cysts of this nature typically originate from the broad ligament. Paraovarian cysts are distinguished by a thin outer wall, measuring less than 3 mm, and are located adjacent to the ovary, but remain separate from it. Typically, on MRI, these structures manifest as simple unilocular structures, exhibiting low SI on T1 images and high SI on T2WI (Fig. 3). However, these structures may contain any type of fluid [16, 17]. A differential diagnosis of paraovarian cyst should include consideration of true ovarian cyst, peritoneal inclusion and hydrosalpinx [17].

#### Dilated fallopian tubes-hydrosalpinx

Hydrosalpinx is a common adnexal lesion that may manifest either as an isolated condition or as a component of a complex pathologic process (e.g. pelvic inflammatory disease, endometriosis, fallopian tube tumour, or tubal pregnancy) that results in distal tubal occlusion. On MRI, hydrosalpinx manifests as a fluid-filled C- or S-shaped tubular structure arising from the upper lateral margin of the uterus [18, 19]. MRI hydrosalpinx with simple fluid shows T2 hyperintense and T1 hypointense signal (Fig. 3), and may contain incomplete septations that correspond to endosalpingeal folds. These enhance after gadolinium injection and should not be considered as solid papillary projections. The thickness of the posterior wall is generally minimal, and this can be augmented by the administration of gadolinium. However, when the dilated tube contains simple fluid but exhibits a thick wall, it is designated as an O-RADS 3 lesion. The presence of internal enhanced solid tissue, such as a mural nodule, should raise suspicion of primary tubal cystadenocarcinoma.

**Table 3:** ORADS 3—MRI characteristics of cystic lesions

Cystic lesion	T2W	T1W	+C T1W	T1Wfs
Unilocular cystic lesions			-	-
Serous ovarian cystadenoma			-	-
Hematosalpinx			-	

### O-RADS MRI 3: cystic ovarian lesions with low risk for malignancy (PPV for malignancy ~5%) (Table 3)

Typically, the O-RADS 3 score is assigned to the following types of cystic adnexal lesions:

#### Unilocular cyst

**Fig. 4:**
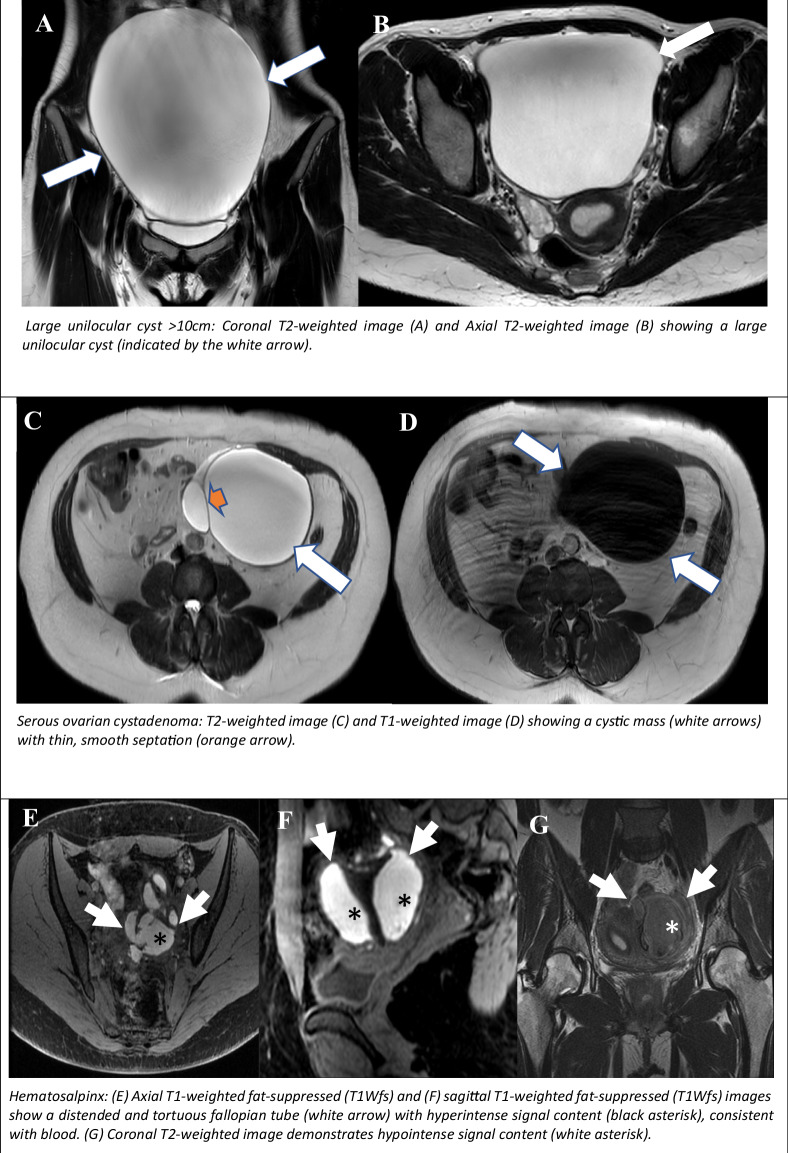
O-RADS MRI 3: cystic ovarian lesions with low risk for malignancy. Large unilocular cyst > 10 cm: coronal T2WI (**A**) and axial T2WI (**B**) showing a large unilocular cyst (indicated by the white arrow). Serous ovarian cystadenoma: T2WI (**C**) and T1-weighted image (**D**) showing a cystic mass (white arrows) with thin, smooth septation (orange arrow). Hematosalpinx: (**E**) axial T1-weighted fat-suppressed (T1Wfs) and (**F**) sagittal T1-weighted fat-suppressed (T1Wfs) images show a distended and tortuous fallopian tube (white arrow) with hyperintense signal content (black asterisk), consistent with blood. **G** The coronal T2WI demonstrates hypointense signal content (white asterisk)

This category encompasses any unilocular ovarian cyst with non-simple fluid, a smooth enhancing wall and without solid tissue. The lesions may contain proteinaceous, haemorrhagic or mucinous fluid. Proteinaceous fluid may be composed of colloid or mucin and may be variable in signal. The SI can be hypointense or intermediate on T2W, and any type of SI on T1W [4], though it typically manifests as a variable high T1W signal [7]. A typical example of such lesions is the non-ruptured ovarian cyst, which manifests as T2 hyperintense and moderate T1 hyperintense SI (Fig. 4). A differential diagnosis of unilocular cyst includes paraovarian cyst, ovarian cystadenoma, serous or mucinous ovarian cystadenoma, and cystic degeneration of subserous or broad ligament leiomyoma.

#### Multilocular cyst

Multilocular cyst is characterised by the presence of smooth, irregular septae and walls with enhancement, and may contain any type of fluid. This category encompasses serous or mucinous ovarian cystadenoma (Fig. 4). A differential diagnosis includes multilocular cyst, serous or mucinous ovarian borderline tumours, which typically exhibit irregular septations. Furthermore, ovarian hyperstimulation syndrome may mimic multilocular ovarian cysts. In such cases, the analysis of the patient’s medical history is crucial in making an accurate diagnosis.

#### Dilated fallopian tubes with no simple fluid

Dilated fallopian tubes with non-simple fluid, yet with a thin enhancing wall, constitute an additional instance of O-RADS MRI score 3 lesions. Such formations frequently contain non-simple fluid, typically of haemorrhagic (non-endometrioid) or proteinaceous nature; they have no solid tissue, with the exception of the endosalpingeal folds. The key sequences utilised for the detection of hematosalpinx are enumerated in Table 2. Hematosalpinx (Fig. 4) is usually associated with tubal pregnancy, pelvic inflammatory disease or even tubal carcinoma. A differential diagnosis includes pyosalpinx associated with TOA. Depending on the viscosity of the pus, the abscess contents may demonstrate heterogeneous restricted diffusion. This procedure can assist in differentiating pus from other types of fluid [20]. However, according to the O-RADS definitions, if the patient presents with acute clinical symptoms and dilated fallopian tubes, the O-RADS MRI score should not be applied. Consequently, pelvic inflammatory disease accompanied by pyosalpinx or TOA is not scored.

**Table 4:** ORADS 4—MRI characteristics of cystic lesions

Cystic lesion	T2W	T1W	+C T1W	T1Wfs
Serous borderline tumour			Papillary enh.	-
Mucinous borderline tumour			Rim/septal enhancement	-
Endodermal sinus tumour of Müllerian origin			Solid enh.	-
Endometrioid borderline ovarian neoplasm			Papillary enh.	
Immature cystic teratoma			Solid enh.	
Teratocarcinoma			Rim/septal/solid enhancement	
Struma ovarii			Rim/septal enhancement	-

### O-RADS MRI 4: cystic ovarian lesions with intermediate risk for malignancy (Table 4), malignancy (PPV for malignancy ~50%)

Typically, the O-RADS 4 score is assigned to the following types of cystic adnexal lesions:

#### Cystic lesions with solid tissue demonstrating an intermediate time-intensity curve on DCE

Serous borderline ovarian tumours (SBOTs) and mucinous borderline ovarian tumours (MBOTs): SBOTs and MBOTs account for 25–30% of “nonbenign” serous tumours [21], and 30–50% of ovarian epithelial borderline tumours [22]. At the time of diagnosis, approximately 70% of SBOTs are confined to one or both ovaries (stage I), while the remaining tumours have “spread” within the pelvis (stage II) or the upper abdomen (stage III). Only rare cases have exhibited an extension beyond the abdomen (stage IV) at the time of diagnosis [23]. SBOTs are defined as cystic tumours with an inner lining composed of polypoid excrescences and pact papillae (endophytic growth). The cystic component may contain serous or dense mucinous fluid. In almost half of the cases, the papillary growth covers the outer surface of the ovary (exophytic growth) [24]. Frequently, both exophytic and endophytic growths may be present. A differential diagnosis of serous and mucinous cystadenocarcinomas is indicated. The hallmark characteristics of carcinoma, such as friability, haemorrhage and necrosis, are typically absent in these tumours [21].

**Fig. 5:**
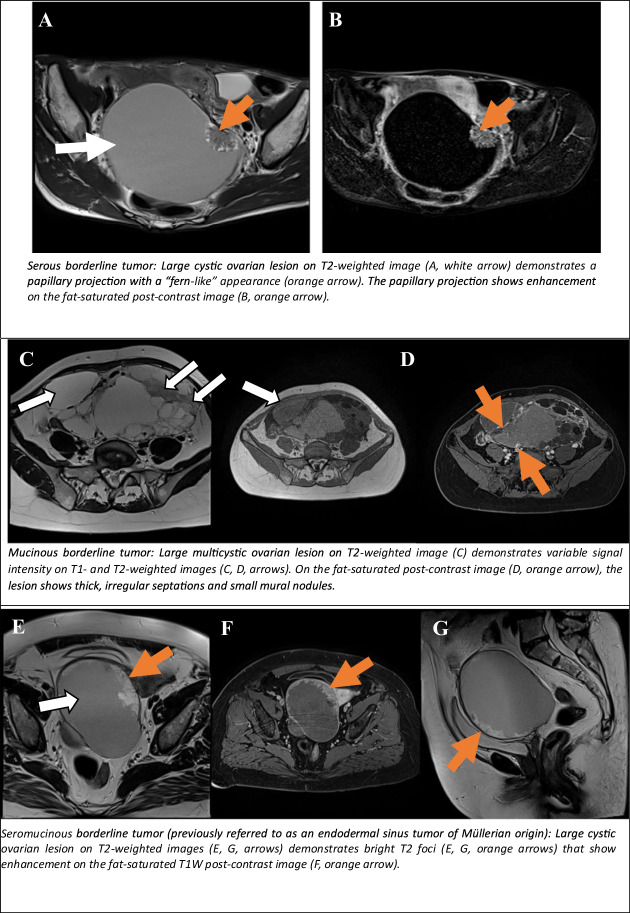

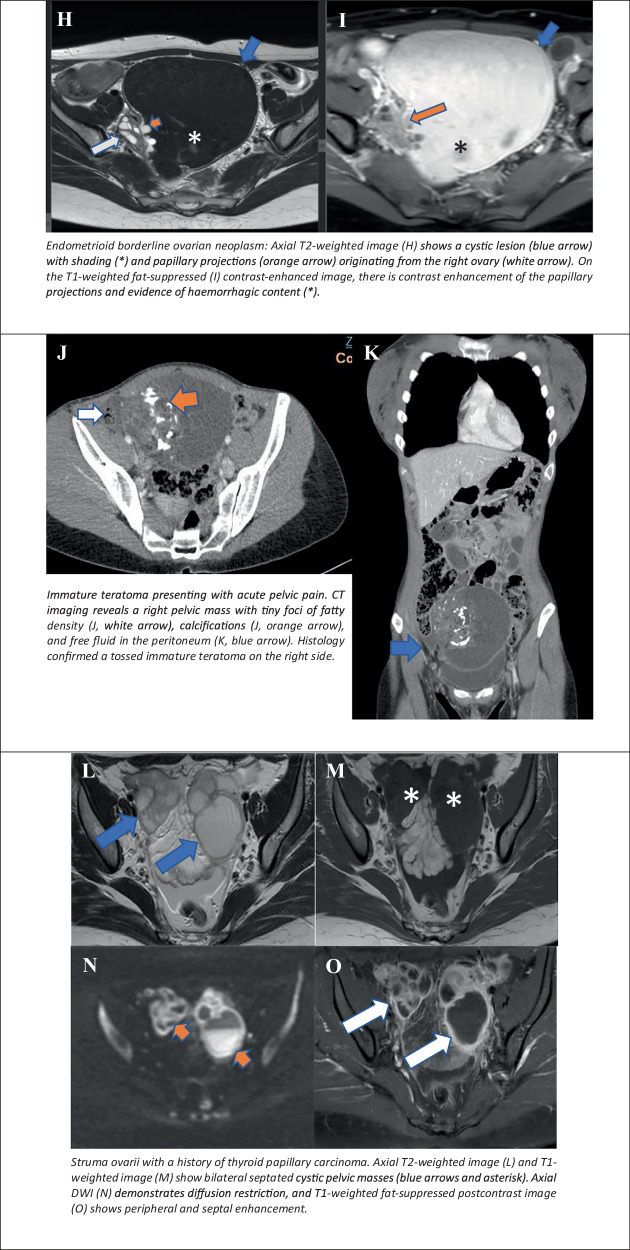
O-RADS MRI 4: cystic ovarian lesions with intermediate risk for malignancy. Serous borderline tumour: large cystic ovarian lesion on T2WI (**A**, white arrow) demonstrates a papillary projection with a “fern-like” appearance (orange arrow). The papillary projection shows enhancement on the fat-saturated post-contrast image (**B**, orange arrow). Mucinous borderline tumour: Large multicystic ovarian lesion on T2WI (**C**) demonstrates variable SI on T1- and T2WI (**C**, **D**, arrows). On the fat-saturated post-contrast image (**D**, orange arrow), the lesion shows thick, irregular septations and small mural nodules. Seromucinous borderline tumour (previously referred to as an endodermal sinus tumour of Müllerian origin): Large cystic ovarian lesion on T2WI (**E**, **G**, arrows) demonstrates bright T2 foci (**E**, **G**, orange arrows) that show enhancement on the fat-saturated T1W post-contrast image (**F**, orange arrow). Endometrioid borderline ovarian neoplasm: Axial T2WI (**H**) shows a cystic lesion (blue arrow) with shading (*) and papillary projections (orange arrow) originating from the right ovary (white arrow). On the T1-weighted fat-suppressed (**I**) contrast-enhanced image, there is contrast enhancement of the papillary projections and evidence of haemorrhagic content (*). Immature teratoma presenting with acute pelvic pain. CT imaging reveals a right pelvic mass with tiny foci of fatty density (**J**, white arrow), calcifications (**J**, orange arrow), and free fluid in the peritoneum (**K**, blue arrow). Histology confirmed a tossed immature teratoma on the right side. Struma ovarii with a history of thyroid papillary carcinoma. Axial T2WI (**L**) and T1-weighted image (**M**) show bilateral septated cystic pelvic masses (blue arrows and asterisk). Axial DWI (**N**) demonstrates diffusion restriction, and the T1-weighted fat-suppressed postcontrast image (**O**) shows peripheral and septal enhancement

MBOTs and Müllerian or endocervical subtype of mucinous borderline tumours usually display more loculi (far more for endocervical subtype), with loculi of different signal intensities, a smaller papillary projection that looks like irregular septation on MRI and a larger size compared to SBOTs (Fig. 5). These tumours accounted for 15% of mucinous borderline tumours [25]. Both MBOTs and M-MBOTs typically manifest as unilateral lesions, exhibiting T1 high SI indicative of haemorrhage or mucinous fluid content, and T2 hypointense signal (Fig. 5). Imaging features suggesting borderline ovarian tumours vs invasive include: the presence of at least one imaging feature suggesting malignancy, the predominance of a cystic-appearing lesion, the absence of mural nodule, the presence of regular thin wall, the presence of normal ipsilateral ovarian stroma, lack of ascites, and the lack of peritoneal and omental cake [26].

Endometrioid borderline ovarian: endometrioid borderline ovarian neoplasms are a rare subtype, accounting for approximately 2–3% of borderline ovarian tumours [27]. These tumours typically manifest as mixed masses with both solid and cystic components. The solid portions may appear as large, solid areas in tumours exhibiting an adenofibromatous pattern or as papillary projections in those with an intracystic growth pattern. According to the literature data, solid components generally exhibit moderately high SI at DWI sequences, irrespective of their growth pattern. Thus, DWI may facilitate the diagnosis of the borderline subtype. Intracystic fluid can exhibit high SI on T1 and low SI on T2 MRI, advocating an association with endometriosis (Fig. 5).

#### Cystic lesion with fat content, but a large volume of enhancing solid tissue

As it was previously mentioned, in contrast to benign teratomas, lipid-containing lesions with more solid elements are usually categorised as O-RADS 4, due to an increased malignant potential.

Malignant transformation of mature cystic teratoma: Malignant transformation of mature cystic teratoma is a rare complication, with a reported incidence of 1–2% of cases [28]. In contrast to mature cystic teratoma, malignant transformation occurs in the 6th or 7th decade of life. The imaging appearance is indicative of the presence of an underlying mature cystic teratoma: a sebaceous lipid component, as well as a heterogeneous, large, solid enhancing component protruding into the cavity or extending transmurally into adjacent organs, corresponding to squamous cell cancer histology [28].

Immature teratoma/teratocarcinoma: immature teratoma and ovarian teratocarcinoma exhibit similar MRI characteristics to other complex cystic-solid adnexal tumours [15] (Fig. 5). Immature teratoma affects younger age groups, usually occurring in the first two decades of life. Immature cystic teratoma may present with analogous MRI characteristics to the mature cystic teratoma; however, it typically contains a smaller amount (droplets) of macroscopic fat. In this instance, the key imaging sequence is T1fs or opposed phase images for the detection of microscopic or intravoxel fat (Fig. 5). The primary distinction between these two entities lies in the presence of a solid component, which is characterised by its larger size and more irregular margins, in the case of immature or suspicious teratoma [10].

Struma ovarii: struma ovarii accounts for 0.3–1% of all ovarian tumours and 3% of all mature teratomas. On MR imaging, the SI of the various solid components varies [29]. The conventional MR imaging appearance of struma ovarii includes the presence of multiple intracystic solid areas, corresponding to thyroid tissue, showing low SI on T2 images and intermediate SI on T1 MRI (Fig. 5). Nevertheless, the cystic spaces demonstrate both high and low SI on T1- and T2WI, suggesting colloid content. In cases of bilateral ovarian lesions and elevated cancer antigen 125 levels, the presence of solid intracystic areas showing high SI on DWI raises suspicion of another malignant process, such as metastatic ovarian disease, including Krukenberg tumour.

### O-RADS MRI 5: cystic ovarian lesions with high risk for malignancy

Lesions classified as O-RADS MRI Score 5 carry a high risk of malignancy, with a malignancy probability of approximately 90% (Table 5). Lesions in this category require urgent evaluation, prompt surgical intervention and further oncological management.

**Table 5:** ORADS 5 - MRI characteristics of cystic lesions

Cystic Lesion	T2W	T1W	+C T1W	T1W fs
Metastasis of colon carcinoma to the ovary			Rim enhancement	
Ductal breast carcinoma metastasis to the left ovary			Rim enhancement	
Ovarian metastasis of endometrial cancer			Solid enhancement	
Low-grade serous ovarian cancer—papillary type			Rim/Papillary enhancement	
Cystic high-grade serous ovarian tumour			Rim/solid enhancement	
Ovarian cystadenocarcinoma			Rim/septal/solid enhancement	
Tubal carcinoma			Rim/nodular enhancement	

Typically, the O-RADS 5 score is assigned to the following types of cystic adnexal lesions:

#### Ovarian metastasis

**Fig. 6:**
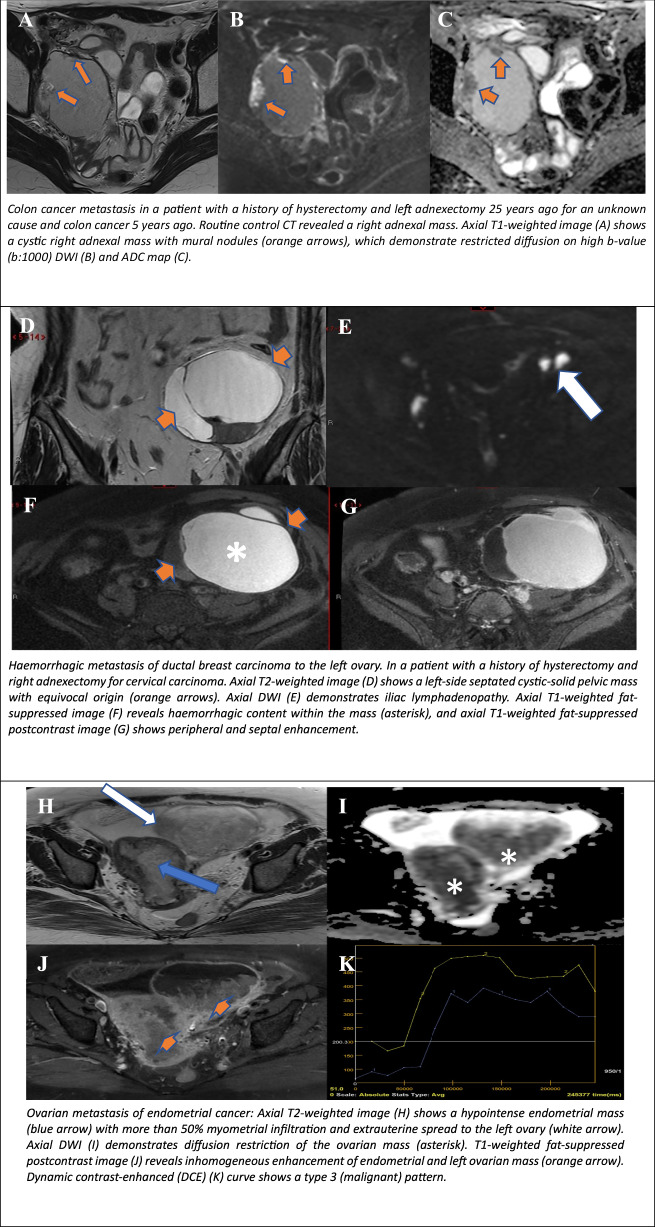

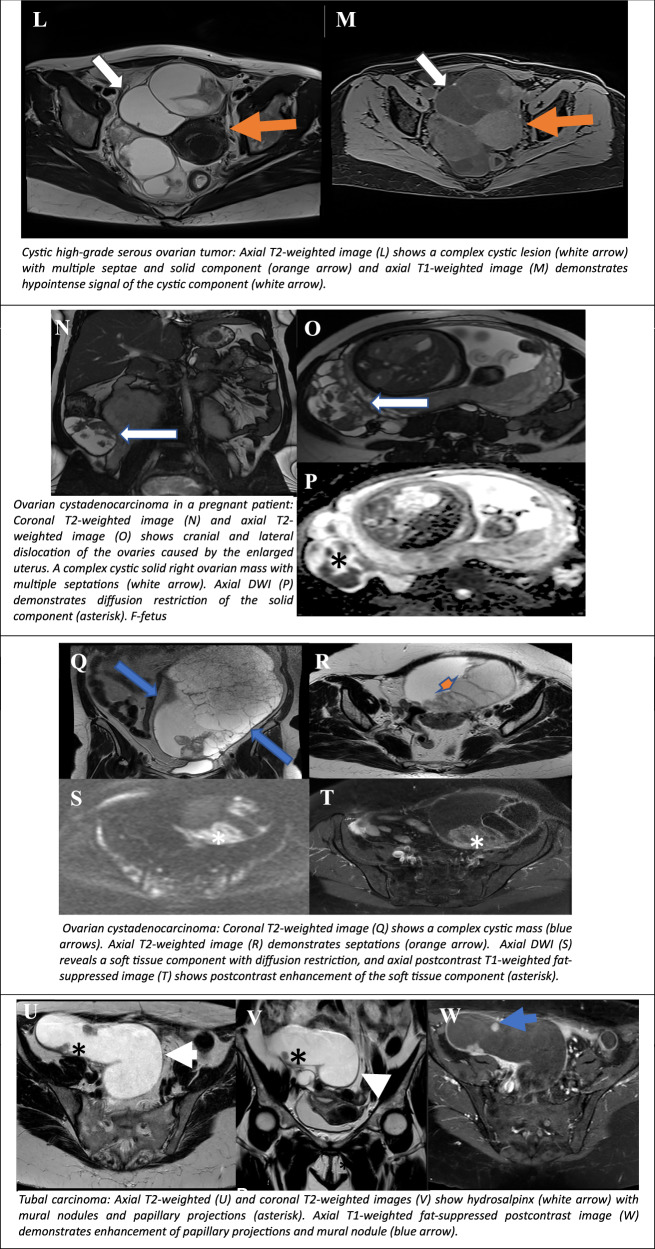
O-RADS MRI 5: cystic ovarian lesions with high risk for malignancy. Colon cancer metastasis in a patient with a history of hysterectomy and left adnexectomy 25 years ago for an unknown cause, and colon cancer 5 years ago. Routine control CT revealed a right adnexal mass. Axial T1-weighted image (**A**) shows a cystic right adnexal mass with mural nodules (orange arrows), which demonstrate restricted diffusion on high b-value (b:1000) DWI (**B**) and ADC map (**C**). Haemorrhagic metastasis of ductal breast carcinoma to the left ovary. In a patient with a history of hysterectomy and right adnexectomy for cervical carcinoma. Axial T2WI (**D**) shows a left-sided septated cystic-solid pelvic mass with equivocal origin (orange arrows). Axial DWI (**E**) demonstrates iliac lymphadenopathy. Axial T1-weighted fat-suppressed image (**F**) reveals haemorrhagic content within the mass (asterisk), and axial T1-weighted fat-suppressed postcontrast image (**G**) shows peripheral and septal enhancement. Ovarian metastasis of endometrial cancer: Axial T2WI (**H**) shows a hypointense endometrial mass (blue arrow) with more than 50% myometrial infiltration and extrauterine spread to the left ovary (white arrow). Axial DWI (**I**) demonstrates diffusion restriction of the ovarian mass (asterisk). T1-weighted fat-suppressed postcontrast image (**J**) reveals inhomogeneous enhancement of the endometrial and left ovarian mass (orange arrow). The dynamic contrast-enhanced (DCE) (**K**) curve shows a type 3 (malignant) pattern. Cystic high-grade serous ovarian tumour: Axial T2WI (**L**) shows a complex cystic lesion (white arrow) with multiple septae and a solid component (orange arrow), and axial T1-weighted image (**M**) demonstrates hypointense signal of the cystic component (white arrow). Ovarian cystadenocarcinoma in a pregnant patient: Coronal T2WI (**N**) and axial T2WI (**O**) show cranial and lateral dislocation of the ovaries caused by the enlarged uterus. A complex cystic solid right ovarian mass with multiple septations (white arrow). Axial DWI (**P**) demonstrates diffusion restriction of the solid component (asterisk). F-fetu. Ovarian cystadenocarcinoma: Coronal T2WI (**Q**) shows a complex cystic mass (blue arrows). Axial T2WI (**R**) demonstrates septations (orange arrow). Axial DWI (**S**) reveals a soft tissue component with diffusion restriction, and axial postcontrast T1-weighted fat-suppressed image (**T**) shows postcontrast enhancement of the soft tissue component (asterisk). Tubal carcinoma: Axial T2-weighted (**U**) and coronal T2WI (**V**) show hydrosalpinx (white arrow) with mural nodules and papillary projections (asterisk). Axial T1-weighted fat-suppressed postcontrast image (**W**) demonstrates enhancement of papillary projections and mural nodule (blue arrow)

The ovaries are a frequent location for metastases from other primaries, including gastric cancer (Krukenberg tumour), uterine (endometrial cancer), cervical, breast, colon, fallopian tube cancer, melanoma and clear cell renal cell carcinoma (ccRCC) [30]. Predominantly solid metastases usually originate from gastric or breast primaries, while other GI tract metastases (e.g. appendiceal, colorectal, and pancreaticobiliary) frequently exhibit larger cystic components. According to the literature, cases of ccRCC metastases are extremely rare, with fewer than 30 documented cases published in the literature [31]. There are only a few reported cases with cystic degeneration [32]. In instances of bilateral ovarian masses with a highly vascular component, the presence of metastases should be considered, particularly in cases of documented history of malignancy. In the context of cystic ovarian metastases, the T2 hypointense signal is heterogeneous due to the varying degrees of cystic degeneration present. Furthermore, solid tissue demonstrates restricted diffusion and a high-risk curve. Another feature indicative of the metastatic nature of the lesion is the preservation of normal ovarian parenchyma, indicating extrinsic invasion (Fig. 6) [9]. The presence of metastases from melanoma is indicated by the presence of haemorrhagic lesions with T1/T1fs hyperintense signal.

#### Serous and mucinous ovarian cystadenocarcinomas

Serous and mucinous ovarian cystadenocarcinomas are complex cystic/solid masses with T2 heterogeneity, thickened irregular septae, a solid component with diffusion restriction and a type 3 curve at DCE MR [33] (Fig. 6). The presence of peritoneal, mesenteric, or omental nodularity or irregular thickening, with or without associated ascites, are features also indicative of malignancy [4].

#### Primary fallopian tube cancer (PFTC)

On MRI, PFTC usually occurs at the distal portion rather than at the proximal portion of the fallopian tube. Since the distal fallopian tube is close to the ovary, PFTC can often be challenging to differentiate from an ovarian tumour. However, the presence of a sausage-shaped complex mass in the adnexal area on MRI may suggest PFTC. The solid lesion of a PFTC demonstrates a homogeneous, relatively T2 hyperintense signal, resembling that of the uterine myometrium. In the context of DWI, the solid component of PFTC appears hyperintense on high b-value images, accompanied by low SI on corresponding ADC maps [34] (Fig. 6).

## Complex adnexal/ovarian lesions that do not score with O-RADS

### TOA

**Fig. 7:**
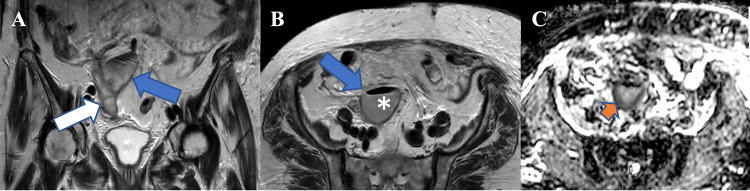
Coronal T2WI (**A**) shows a TOA (blue arrow) and a dilated tube filled with fluid (white arrow) in the projection of the right ovary, while axial T2WI (**B**) demonstrates the abscess (blue arrow) with a gas-fluid level (asterisk), and DWI image (**C**) reveals peripheral diffusion restriction (orange arrow)

TOA is defined as the accumulation of frank pus or an inflammatory mass within the fallopian tube and ovary. TOA typically manifests as a complication of pelvic inflammatory diseases in young women, although it can occasionally occur in post-menopausal patients as well. The presence of air locules within the cystic lesion is a strong indicator of abscess formation. Ovarian abscess demonstrates a thick, irregular wall, internal septations, an air-fluid level and surrounding fatty infiltration/stranding. TOAs typically manifest low SI on T1W and heterogeneously high SI on T2W [13] (Fig. 7).

## Non-ovarian cystic female pelvic lesions

### Peritoneal inclusion cyst (PIC)

PIC is a relatively common pelvic cystic lesion, primarily encountered in reproductive-age women, resulting from chronic peritoneal irritation. While typically benign, some authors suggest PIC may have neoplastic potential due to high recurrence rates and rare malignant transformation [35, 36].

PIC is usually asymptomatic, although in cases of substantial lesions, symptoms may manifest as chronic pelvic pain, dyspareunia, constipation, or urinary dysfunction.

**Fig. 8:**
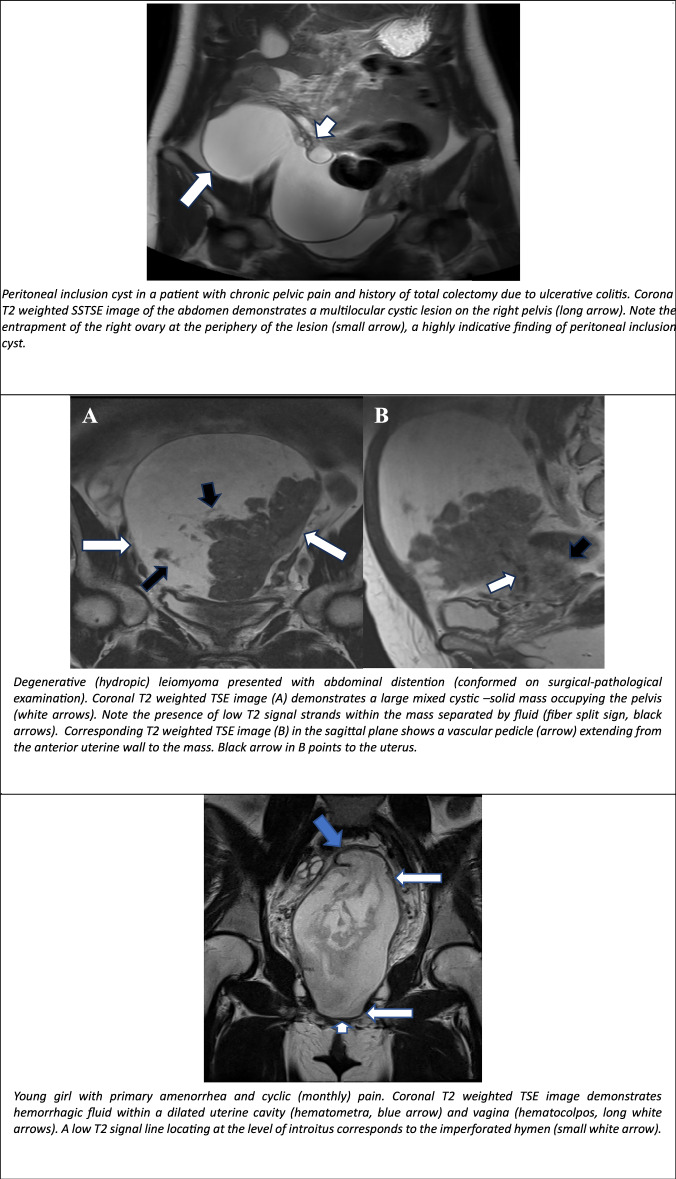

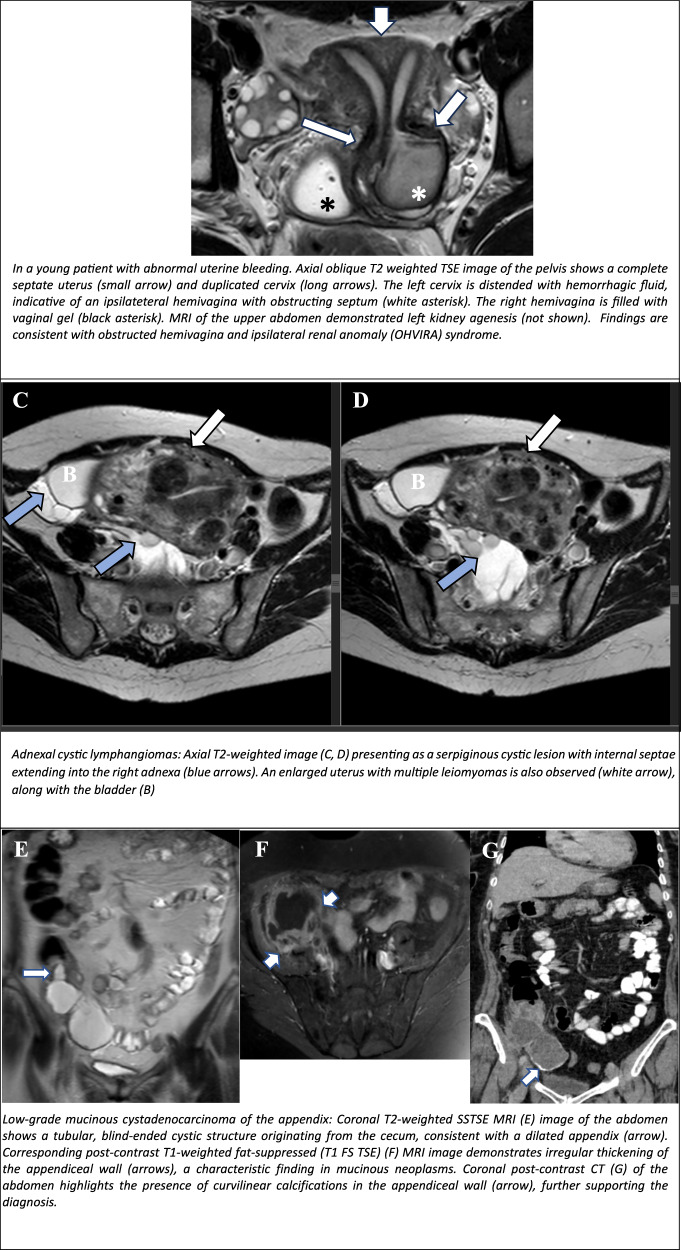
Non-ovarian cystic female pelvic lesions. PIC in a patient with chronic pelvic pain and a history of total colectomy due to ulcerative colitis. Coronal T2-weighted SSTSE image of the abdomen demonstrates a multilocular cystic lesion on the right pelvis (long arrow). Note the entrapment of the right ovary at the periphery of the lesion (small arrow), a highly indicative finding of PIC. A degenerative (hydropic) leiomyoma presented with abdominal distention (confirmed on surgical-pathological examination). Coronal T2-weighted TSE image (**A**) demonstrates a large mixed cystic–solids mass occupying the pelvis (white arrows). Note the presence of low T2 signal strands within the mass separated by fluid (fibre split sign, black arrows). Corresponding T2-weighted TSE image (**B**) in the sagittal plane shows a vascular pedicle (arrow) extending from the anterior uterine wall to the mass. The black arrow in (**B**) points to the uterus. Young girl with primary amenorrhoea and cyclic (monthly) pain. Coronal T2-weighted TSE image demonstrates haemorrhagic fluid within a dilated uterine cavity (hematometra, blue arrow) and vagina (hematocolpos, long white arrows). A low T2 signal line located at the level of introitus corresponds to the imperforated hymen (small white arrow). In a young patient with abnormal uterine bleeding. Axial oblique Τ2 weighted TSE image of the pelvis shows a complete septate uterus (small arrow) and duplicated cervix (long arrows). The left cervix is distended with haemorrhagic fluid, indicative of an ipsilateral hemivagina with an obstructing septum (white asterisk). The right hemivagina is filled with vaginal gel (black asterisk). MRI of the upper abdomen demonstrated left kidney agenesis (not shown). Findings are consistent with obstructed hemivagina and ipsilateral renal anomaly (OHVIRA) syndrome. Αdnexal cystic lymphangiomas: axial T2WI (**C**, **D**) presenting as a serpiginous cystic lesion with internal septae extending into the right adnexa (blue arrows). An enlarged uterus with multiple leiomyomas is also observed (white arrow), along with the bladder (**B**). Low-grade mucinous cystadenocarcinoma of the appendix: Coronal T2-weighted SSTSE MRI (**E**) image of the abdomen shows a tubular, blind-ended cystic structure originating from the caecum, consistent with a dilated appendix (arrow). Corresponding post-contrast T1-weighted fat-suppressed (T1 FS TSE) (**F**) MRI image demonstrates irregular thickening of the appendiceal wall (arrows), a characteristic finding in mucinous neoplasms. Coronal post-contrast CT (**G**) of the abdomen highlights the presence of curvilinear calcifications in the appendiceal wall (arrow), further supporting the diagnosis

On MRI, PIC appears as a multiloculated, non-enhanced wall, fluid-filled pelvic lesion with variable septal thickness. It often contains serous fluid, debris, or a haemorrhage. The distinctive “spider-in-web” configuration, in which the ovaries are entrapped within the cyst, strongly suggests PIC (Fig. 8). T2W MRI shows a hyperintense lesion, while T1WI may display high SI in the presence of haemorrhage. Contrast-enhanced imaging reveals variable septal enhancement, thus aiding in the differentiation from other pelvic masses [1].

### Degenerative (hydropic) leiomyoma

Degenerative changes are prevalent in uterine leiomyomas, affecting approximately 10% of cases. Hydropic leiomyoma, recognised as a distinct WHO subtype, is characterised by zonal watery oedema. Extensive fluid accumulation can lead to large, predominantly cystic tumours, making differentiation from cystic ovarian tumours, especially pedunculated ones, challenging [37, 38].

Typical MRI features of hydropic leiomyomas include a large, well-circumscribed mass with high T2 SI, reflecting abundant watery oedema, and a few enhancing cord-like components with low T2 signal, corresponding to solid tumour portions [39] (Fig. 8). Intratumoural flow voids, indicative of thick-walled vessels, are common, while haemorrhagic or necrotic areas are rare. Although tumour markers are normal in degenerative leiomyomas, slight elevations may occur due to peritoneal irritation from abdominal overdistension [40–43].

### Congenital uterine/vaginal malformations mimicking cystic pelvic masses

Obstructive anomalies impede normal menstrual outflow, resulting in blood accumulation within the uterus and/or vagina, increasing the risk of retrograde menstruation and endometriosis. These conditions predominantly affect adolescents and young women, presenting with amenorrhoea, dysmenorrhoea, and pelvic pain. Dilatation of the uterovaginal tract due to retained blood may mimic cystic pelvic tumours, thus necessitating a detailed history, examination, and imaging for accurate diagnosis [44–46].

MRI is superior to US in identifying the level of obstruction. In the case of an imperforate hymen, the vagina is distended (hematocolpos), appearing hyperintense on T1W MRI, with a variable T2W signal depending on blood evolution. A low T2-intensity line at the introitus is indicative of the hymen (Fig. 8). A Transverse vaginal septum appears as a hypointense line relative to the blood-filled vagina, typically located in the middle or upper third of the vagina. In contrast, an obstructed longitudinal vaginal septum is associated with uterine didelphys or septate uterus (Fig. 8). MRI evaluates septal thickness and location and detects hematometra or hematosalpinx. If the fluid-filled vaginal cavity is disproportionately larger than the uterus, a vaginal septum should be suspected.

### Αdnexal cystic lymphangiomas

Lymphangiomas are rare, benign tumours of the lymphatic system, consisting of endothelial-lined cystic spaces filled with serous or chylous fluid. Their precise aetiology of this condition in adults remains unclear [47].

Less than 5% of lymphangiomas manifest in the abdomen, with the adnexal locations, involving the ovaries, fallopian tubes, or paraovarian space, accounting for less than 1% of all abdominal cases. The definitive diagnosis relies upon histopathological examination [48].

On MRI, lymphangiomas typically manifest as unilocular or multilocular serpiginous cystic masses, encasing, though not obstructing, the ovarian vein as it extends caudally around the ipsilateral ovary or cranially into the retroperitoneal space. This relationship with ovarian vessels is a highly specific imaging feature of adnexal cystic lymphangiomas, distinguishing them from other cystic adnexal lesions (Fig. 8) [49, 50].

Cystic fluid may demonstrate low (serous) or high (chylous) SI on T1-weighted MRI, and no internal solid enhancing components are present. Due to their potential to mimic ovarian cystic lesions, long-term follow-up may be required due to rare malignant transformation [50, 51].

### Mucocele of the appendix

Mucoceles are a rare occurrence, with an incidence of 0.2–0.3%, predominantly affecting females (4:1 ratio) of menopausal age [52]. Appendiceal mucocele results from abnormal appendix distension due to luminal obstruction, which can be non-neoplastic (e.g. mucosal hyperplasia, retention cysts, appendicoliths, endometriosis) or neoplastic, with primary appendiceal tumours accounting for ~1% of appendectomy specimens [53, 54].

The 2010 WHO classification delineates mucinous adenomas, low-grade appendiceal mucinous neoplasms (LAMNs), and mucinous adenocarcinomas. Pseudomyxoma peritonei (PMP) is categorised as either low-grade PMP (LAMN-associated) or high-grade PMP (adenocarcinoma-associated) [55].

The MRI findings include the presence of a tubular cystic mass in the right lower quadrant, hyperintense on T2WI, with variable T1 SI (Fig. 8). Restricted diffusion on DWI is more prevalent in malignant lesions, while a stratified “onion skin” sign may manifest in mucin-rich lesions. Post-contrast imaging reveals mild to moderate enhancement of the vessel wall, raising suspicion for neoplastic transformation [53].

### Tailgut cyst

Tailgut cysts are rare congenital anomalies arising from primitive hindgut remnants in the retrorectal-presacral space [56]. These lesions are more prevalent among women and can appear at any age, are often asymptomatic, and are detected incidentally on CT or MRI.

**Fig. 9:**
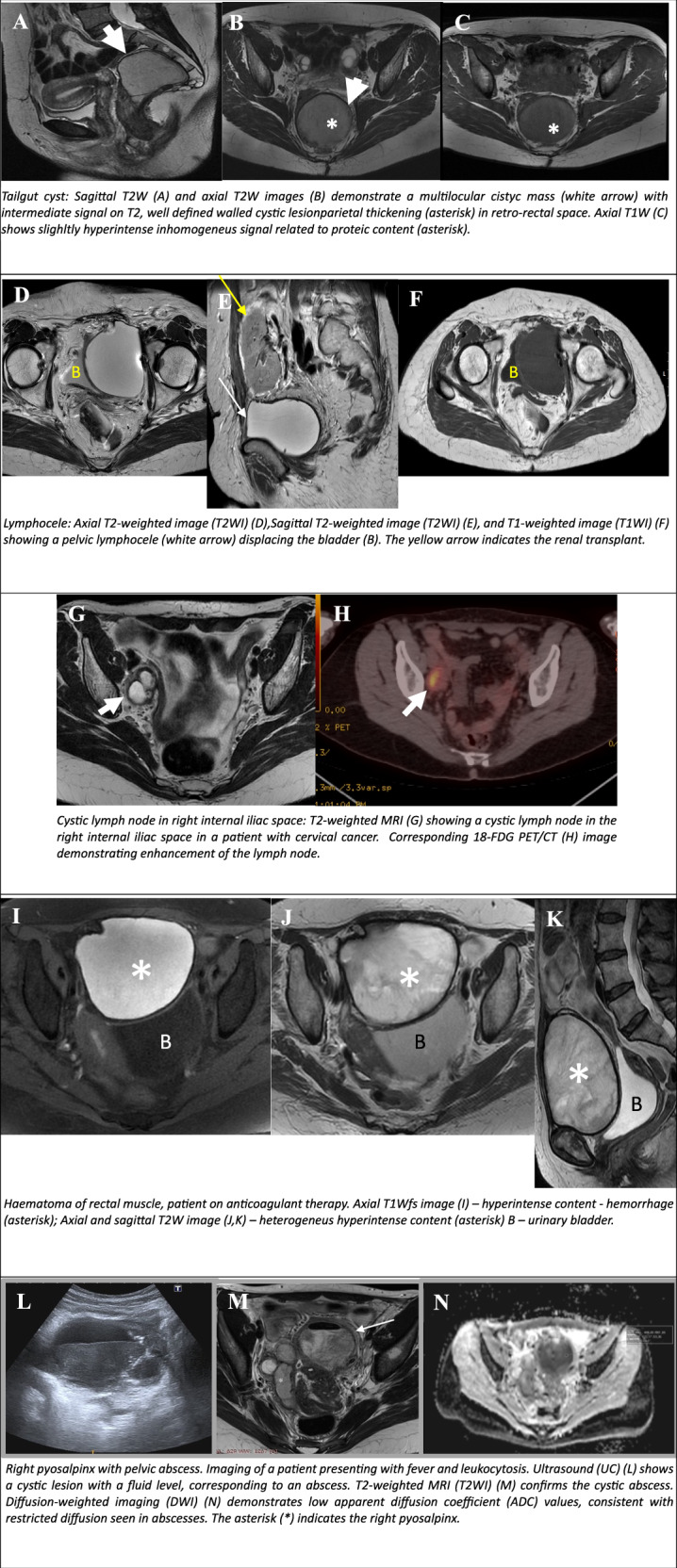
Non-ovarian cystic female pelvic lesions. Tailgut cyst: sagittal T2W (**A**) and axial T2W images (**B**) demonstrate a multilocular cystic mass (white arrow) with intermediate signal on T2, a well-defined walled cystic lesion with parietal thickening (asterisk) in the retro-rectal space. Axial T1W (**C**) shows a slightly hyperintense inhomogeneous signal related to proteic content (asterisk). Lymphocele: axial T2WI (T2WI) (**D**), sagittal T2WI (T2WI) (**E**), and T1-weighted image (T1WI) (**F**) showing a pelvic lymphocele (white arrow) displacing the bladder (**B**). The yellow arrow indicates the renal transplant. Cystic lymph node in right internal iliac space: T2-weighted MRI (**G**) showing a cystic lymph node in the right internal iliac space in a patient with cervical cancer. Corresponding 18-FDG PET/CT (**H**) image demonstrating enhancement of the lymph node. Haematoma of the rectal muscle, patient on anticoagulant therapy. Axial T1Wfs image (**I**)—hyperintense content—haemorrhage (asterisk); axial and sagittal T2W image (**J**, **K**)—heterogeneous hyperintense content (asterisk) **B**—urinary bladder. Right pyosalpinx with pelvic abscess. Imaging of a patient presenting with fever and leukocytosis. Ultrasound (UC) (**L**) shows a cystic lesion with a fluid level, corresponding to an abscess. T2-weighted MRI (T2WI) (**M**) confirms the cystic abscess. Diffusion-weighted imaging (DWI) (**N**) demonstrates low apparent diffusion coefficient (ADC) values, consistent with restricted diffusion seen in abscesses. The asterisk (*) indicates the right pyosalpinx

MRI is the preferred modality, with these cysts manifesting as uni- or multilocular, thin-walled lesions filled with mucoid material, typically located in the retrorectal space. On MRI, they exhibit high to intermediate T2 signal and variable T1 signal, depending on contents present, including mucin, protein, fat, or haemorrhage (Fig. 9).

Complications include infection, leading to abscess formation, or malignant transformation [57]. Features suggestive of malignancy include solid components with DWI restriction and contrast uptake, irregular walls, lymphadenopathy, or direct invasion.

Differential diagnoses include other presacral cystic masses such as epidermoid cysts, dermoid cysts, cystic lymphangiomas, or anterior meningoceles [58].

### Lymphocele

A lymphocele is defined as a lymphatic-filled cystic lesion, usually after systematic and/or para-aortic lymphadenectomy. The incidence of lymphocele following pelvic lymph node (LN) dissection for patients with gynaecological malignancies ranges from 1% to 58% [59].

On imaging, lymphoceles are typically depicted as unilocular, thin-walled, fluid-filled structures, unless complicated by infection or haemorrhage (Fig. 9). It is imperative to distinguish between lymphocele and other postoperative complications, such as haematoma, seroma, abscess, and cystic tumour recurrence [60].

Usually, they are detected during routine follow-up procedures. Only 5–18% of lymphoceles present with symptoms, including pelvic pain, infection, or symptoms related to compression of adjacent structures. Only symptomatic lymphoceles should be treated with US or CT-guided percutaneous drainage, possibly accompanied by sclerotisation.

### Cystic degeneration of LNs

Cystic or necrotic appearing LNs can be caused by several infectious, inflammatory or malignant conditions, including metastatic LNs from squamous cell carcinoma that originates from the genitalia [1].

On imaging (CT, MRI), these appear as thin-walled, cystic LNs located along the typical anatomical line of the LNs' location. This distinction is crucial for differentiating them from ovarian masses (Fig. 9).

### Pelvic haematoma

Acute pelvic haematomas are typically characterised by hyperattenuation on non-enhanced computed tomography, and the presence of a fluid–fluid level may suggest active bleeding. After 2–3 weeks, it becomes hypoattenuating due to liquefaction, and manifests as a simple cyst with water attenuation. Chronic haematomas may present a rim of calcification. At MR imaging, acute and subacute haematomas exhibit high SI on T1-weighted fat-suppressed images. Subsequently, a thick, dark peripheral rim may be seen on both T1- and T2WI, accompanied by a bright inner ring on T1-weighted images, a finding known as the concentric ring sign (Fig. 9). Haematoma does not enhance with the administration of contrast material. Subtraction images are useful in ruling out the presence of wall solid nodules and/or septa that may be seen in pelvic masses with haemorrhage. A further differential diagnosis is that of endometriomas, which are diagnosed in the appropriate clinical context [61].

### Abscess

Pelvic abscess is a serious complication of lower genital tract infections, including pelvic inflammatory disease, postoperative infections following pelvic instrumentation or extragenital causes such as inflammatory bowel disease, diverticulitis, and appendicitis. Furthermore, non-infected collections such as haematomas or lymphoceles, may progress to abscesses in the event of secondary infection.

The MRI is valuable to ascertain the extent of the disease, particularly when ultrasound findings are inconclusive. DWI adds significant diagnostic value in detecting, characterising, and monitoring abscesses. Abscess typically manifests restricted diffusion because of the high cellularity and viscous purulent material within the structure. Facilitating to distinction of abscesses from cystic or necrotic tumours, which usually do not restrict diffusion as strongly [62] (Fig. 9).

### Cystic schwannoma

Schwannomas are mostly solid and benign encapsulated tumours arising from Schwann cells that compose the peripheral nerve sheath. The tumours are typically solitary and asymptomatic. In larger sizes, they may compress peripheral nerve structures, leading to radiating pain or specific neurologic deficits in the distribution of the nerve origin [63, 64].

**Fig. 10:**
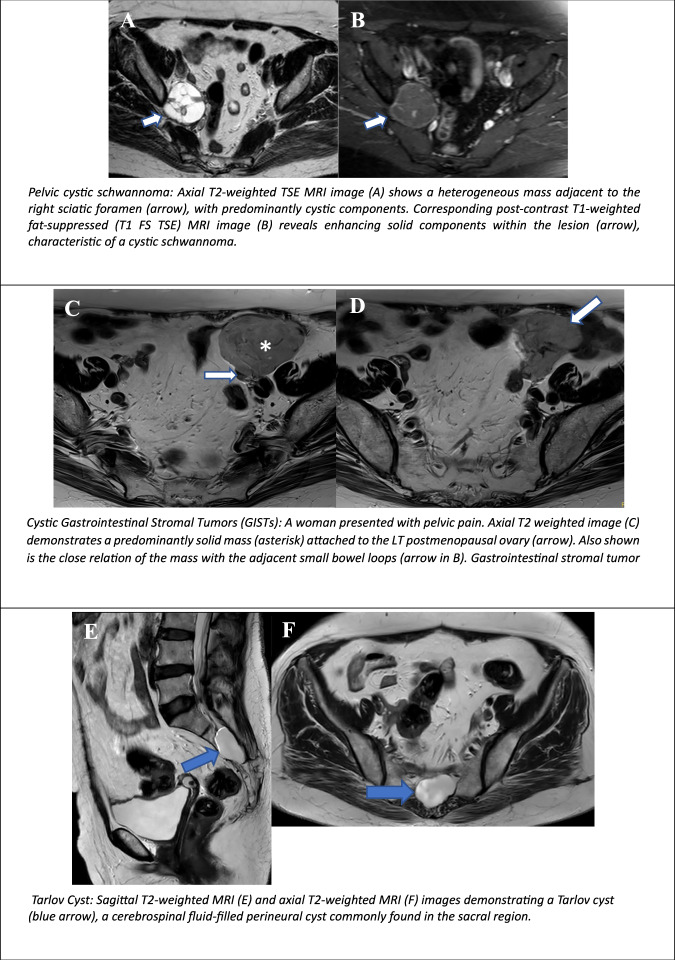

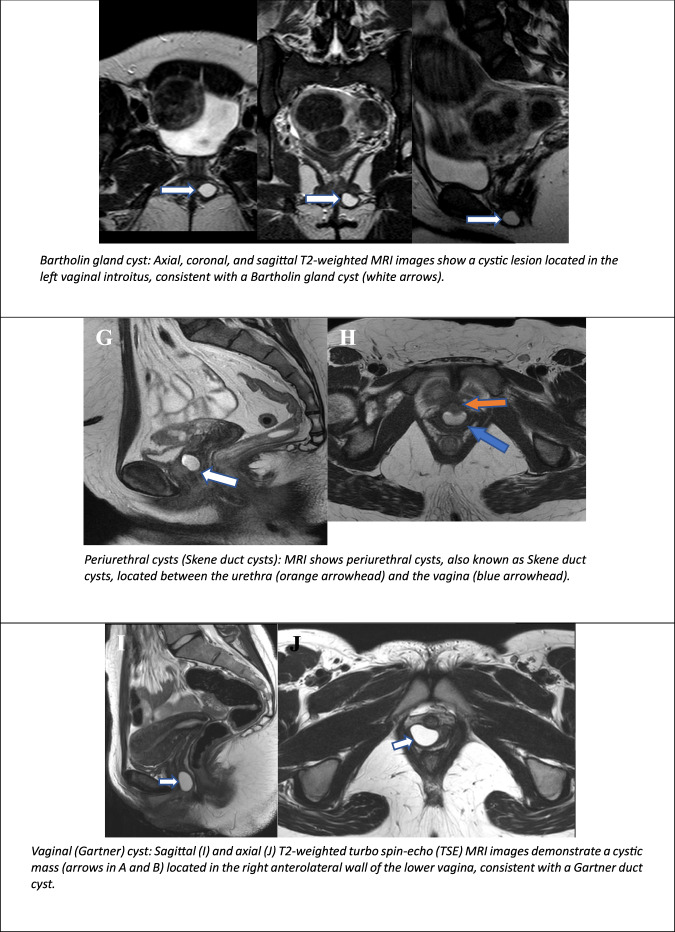
Non-ovarian cystic female pelvic lesions. Pelvic cystic schwannoma: Axial T2-weighted TSE MRI image (**A**) shows a heterogeneous mass adjacent to the right sciatic foramen (arrow), with predominantly cystic components. Corresponding post-contrast T1-weighted fat-suppressed (T1 FS TSE) MRI image (**B**) reveals enhancing solid components within the lesion (arrow), characteristic of a cystic schwannoma. Cystic Gastrointestinal Stromal Tumours (GISTs): a woman presented with pelvic pain. Axial T2WI (**C**) demonstrates a predominantly solid mass (asterisk) attached to the LT postmenopausal ovary (arrow). Also shown is the close relation of the mass with the adjacent small bowel loops (arrow in **D**). Gastrointestinal stromal tumour Tarlov Cyst: Sagittal T2-weighted MRI (**E**) and axial T2-weighted MRI (**F**) images demonstrating a Tarlov cyst (blue arrow), a cerebrospinal fluid-filled perineural cyst commonly found in the sacral region. Bartholin gland cyst: axial, coronal, and sagittal T2-weighted MRI images show a cystic lesion located in the left vaginal introitus, consistent with a Bartholin gland cyst (white arrows). Periurethral cysts (Skene duct cysts): MRI shows periurethral cysts, also known as Skene duct cysts (**G**), located between the urethra (orange arrowhead) and the vagina (blue arrowhead) (**H**). Vaginal (Gartner) cyst: sagittal (**I**) and axial (**J**) T2-weighted turbo spin-echo (TSE) MRI images demonstrate a cystic mass (arrows in **I**, **J**) located in the right anterolateral wall of the lower vagina, consistent with a Gartner duct cyst

On imaging, these lesions manifest a fusiform appearance, and the so-called “split-fat sign”, whereby the lesion is surrounded by a rim of fat due to the displacement of the fat that normally surrounds the neurovascular bundle. On MRI, the low-signal margin can also be seen on T1 and T2W; this margin corresponds to the epineurium surrounding the schwannoma (Fig. 10).

Schwannomas can undergo degenerative changes, including cyst formation, calcification, haemorrhages, and fibrosis. A purely cystic appearance is rare in schwannomas, and these masses can be described as benign cyst-like solids or partly solid lesions based on the degree of degenerative changes.

### Cystic gastrointestinal stromal tumours (GISTs)

Gastrointestinal stromal tumours (GISTs) account for 0.1–3% of all gastrointestinal tumours. The location of these tumours is typically in the stomach, followed by the small bowel or colon. The majority of the GISTs arise from the muscularis propria of the GI tract, and are frequently exophytic, extending into the lower abdomen and pelvis (Fig. 10). However, they can also, albeit less frequently, arise from organs outside the luminal gastrointestinal tract (extra-gastrointestinal stromal tumour, EGIST) mimicking gynaecological neoplasms such as pedunculated fibroids or ovarian tumours. Imaging features demonstrate variability, ranging from homogeneous solid masses to heterogeneous solid masses with internal areas of cystic degeneration/necrosis or haemorrhage. However, it is considered unusual for this to manifest as predominant cystic neoplasms. Pelvic ultrasound findings are non-specific, particularly in cases of large tumours. In such cases, CT or MRI may be required to detect any association with the GI tract [65, 66].

### Mesenteric cysts

Mesenteric cysts are rare lesions defined as any cyst arising in the mesentery. The dimensions of a mesenteric cyst may vary from a few centimetres to a large mass occupying the entire abdomen and pelvis. Consequently, these cysts should be considered in the differential diagnoses of the cystic pelvic neoplasms in female patients. The majority of cysts are asymptomatic and are usually detected incidentally; common symptoms include abdominal pain, distension or a palpable abdominal mass. Further complications may include torsion, bleeding, or even rupture. Imaging findings include the presence of a large unilocular or multilocular cystic mass with variable wall thickness and cystic content, related to the histology of the lesion. Typically, simple cysts contain serous fluid, while chlyolymphatic cysts may demonstrate a fluid-fluid level due to the presence of chyle. At MRI, the fat content of the chyle manifests an increased signal on T1 and T2 images, accompanied by a signal drop on T1-weighted out-of-phase and fat-suppressed images. In the majority of cases, surgical removal of the cyst is necessary, and histopathological analysis is required to establish a definitive diagnosis [35].

### Tarlov cyst

Tarlov cysts (or perineural cysts) are defined as dilatations of the arachnoid and dura of the spinal posterior nerve root sheath, which contains the nerve fibres. These malformations can occur in any of the regions along the spine, although they are more prevalent within the lower lumbar spine and sacrum, especially S2 and S3 nerve roots. Typically, Tarlov cysts manifest as thin-walled cystic lesions without enhancement, which may or may not cause neural foraminal widening or bone remodelling [67]. Substantial lesions may mimic adnexal cystic tumours on US. MRI can accurately identify the origin of the lesion and its association with the nerve sheath. On T1WI and T2W1 sequences, these lesions are well-circumscribed, intra/transforaminal, and are isointense to cerebrospinal fluid without evidence of enhancement [68] (Fig. 10).

### Urethral, nabothian, vaginal and bartholin cysts

The Bartholin gland, derived from the urogenital sinus, can be considered the female analogue of the male Cowper’s (bulbourethral) gland. It is located posterior to the vaginal introitus, inferior to the perineal membrane, and is the most common site for vulvar cysts or abscesses, occurring in up to 2% of women. Ductal obstruction leads to cyst formation, which may progress to an abscess if infected [69] (Fig. 10).

The Skene glands, analogous to the male prostate, are periurethral glands located at the 3 and 9 o’clock positions along the distal urethra. Cysts in these glands may extend along the external urethral orifice and, when enlarged, can cause obstruction or infection. On MRI, they manifest as round or ovoid structures, situated laterally to the external urethral orifice, inferior to the pubic symphysis, and may contain proteinaceous or haemorrhagic material, exhibiting variable T1- and T2-weighted SI. Typically, these cysts are characterised by thin, non-enhancing cystic walls [70] (Fig. 10).

Nabothian cysts are mucus-filled cysts that form on the cervix. Such cysts are formed when the openings of the cervical glands become blocked, resulting in mucus accumulation and subsequent cyst formation. They are commonly benign and often asymptomatic. On US and MRI scans, the cysts manifest as small, smooth, rounded protuberances.

Gartner duct cysts are the most prevalent benign cysts along the anterolateral wall of the upper vagina, manifesting in 1% of women. These structures are formed from the remnants of the distal mesonephric ducts. Typically, they are associated with other congenital urogenital abnormalities, such as unilateral renal agenesis, hypoplasia or ectopic ureteral insertion. They can also have variable SI on T1 depending on the proteinaceous content within cyst fluid, and the potential existence of thin septa has been indicated [69, 70] (Fig. 10).

## Conclusion

Cystic female pelvic lesions encompass a broad spectrum of pathological entities, ranging from benign physiological cysts to malignant neoplasms. Given their overlapping imaging characteristics, precise diagnosis can be challenging. MRI is of particular importance when utilising the O-RADS MRI risk stratification system, as it enables the differentiation between benign and malignant lesions, thus guiding clinical decision-making and optimising patient management.

## Data Availability

Availability of data and materials are contained in the Supplementary material.

## Abbreviations

LN: Lymph node
MBOT: Mucinous borderline ovarian tumour
O-RADS: Ovarian-adnexal reporting and data system
PFTC: Primary fallopian tube cancer
PIC: Peritoneal inclusion cyst
PMP: Pseudomyxoma peritonei
SBOT: Serous borderline ovarian tumour
SI: Signal intensity
TOA: Tubo-ovarian abscess
